# Brown adipose tissue in ruminants: development, thermogenic function, and nutritional regulation

**DOI:** 10.3389/fvets.2026.1819034

**Published:** 2026-05-15

**Authors:** Zhuo-Hang Xiao, Hong-Wei Duan, Lian Li

**Affiliations:** College of Animal Science and Technology, Nanjing Agricultural University, Nanjing, China

**Keywords:** brown adipose tissue, energy metabolism, nutritional regulation, ruminants, thermogenesis

## Abstract

Brown adipose tissue (BAT) is essential for neonatal ruminants to maintain body temperature and survive postnatal cold stress through non-shivering thermogenesis. At birth, BAT is abundant and mainly distributed in the neck, shoulder, perirenal region, and around the heart, but declines with age (“whitening”). This review summarizes BAT developmental dynamics, physiological functions, and the nutritional regulation of BAT, focusing on how fatty acids, carnitine, vitamins, and minerals modulate thermogenic activity through effects on mitochondrial function, lipid metabolism, and key signaling pathways (e.g., *UCP1, PGC-1*α, and *PPAR*γ). Evidence indicates that targeted nutritional strategies can enhance BAT function, improving neonatal cold tolerance, survival, and early growth performance. Finally, we highlight the use of multi-omics technologies as a promising future direction to decipher BAT regulatory networks and guide precise nutritional interventions.

## Highlights

- Brown adipose tissue (BAT) is a key thermogenic tissue in ruminants, particularly abundant in newborns, and plays an essential role in non-shivering thermogenesis and early-life cold adaptation.
- This review systematically summarizes the distribution, developmental dynamics, physiological functions, and molecular regulatory mechanisms of BAT in ruminant species.
- Nutritional regulation of BAT through specific nutrients offers promising opportunities to enhance thermoregulation, stress resistance, and production performance in ruminant livestock.

## 1 Introduction

Adipose tissue in animals can be broadly classified into white adipose tissue (WAT), brown adipose tissue (BAT), and beige adipose tissue, which differ markedly in anatomical distribution, cellular morphology, and physiological function (1). Among these, BAT is a specialized thermogenic tissue composed of small adipocytes with multiple lipid droplets and abundant mitochondria, serving as the primary site of non-shivering thermogenesis (NST) in young ruminants (2). By oxidizing lipids and carbohydrates, BAT plays a critical role in energy metabolism and body temperature regulation (3). The thermogenic function of BAT is mainly mediated by uncoupling protein 1 (*UCP1*), located in the inner mitochondrial membrane, which dissipates the proton gradient generated during oxidative phosphorylation as heat rather than ATP (4, 5). This mechanism is essential for neonatal ruminants to cope with postnatal cold stress. Immediately after birth, calves and lambs are exposed to a cold extrauterine environment, making BAT-mediated NST crucial for thermoregulation, survival, and early postnatal development (6–8). Accordingly, BAT depots in the interscapular, cervical, and perirenal regions represent the principal heat-producing tissues during early life (7, 9). Accumulating evidence suggests that both BAT mass and thermogenic activity are positively associated with cold tolerance, survival rate, and early growth performance in neonatal ruminants (10, 11). In cold climates, neonates exhibiting higher BAT activity display superior cold resistance and survival advantages (12, 13). In calves, BAT is primarily distributed in the interscapular and perirenal regions, where adipocytes are enriched in mitochondria and express high levels of *UCP1*, conferring strong thermogenic capacity (8, 13). In lambs, Alexander et al. conducted macroscopic dissections of several breeds of newborns and found that the distribution of brown adipose tissue was largely consistent with that reported for other ruminant species. BAT accounted for approximately 1.5% of body weight and was primarily located in the perirenal abdominal and prescapular cervical regions (14).

Most current studies in ruminants, particularly in cattle, have provided the primary basis for understanding BAT biology (13, 15). Traditionally, BAT has been assumed to undergo progressive “whitening” with advancing age as ruminants develop insulating hair coats, alter body fat composition, and achieve rumen functional maturity, resulting in a marked decline in thermogenic activity (16). However, recent studies indicate that under prolonged cold exposure or specific nutritional interventions, thermogenically competent beige adipocytes can emerge within WAT, a process termed adipose tissue browning, highlighting the considerable plasticity of ruminant adipose tissue (8, 15, 16). This finding highlights the previously underestimated plasticity of ruminant adipose tissue. For example, cold exposure has been shown to induce browning of subcutaneous WAT in cattle, characterized by reduced adipocyte size, increased expression of browning markers, and a metabolic shift toward lipid catabolism (17). Beyond environmental stimuli, nutritional regulation represents one of the most practical approaches for modulating BAT development and function (18). Specific nutrients, including fatty acids, vitamins, and minerals, can regulate BAT activity through diverse molecular pathways (19).

A comprehensive understanding of BAT biology and its regulatory mechanisms in ruminants is of substantial theoretical and practical significance. From a production standpoint, high early-life mortality in calves and lambs remains a major challenge for livestock systems, with cold stress constituting a critical environmental risk factor (20). Targeted nutritional regulation of BAT development and thermogenic function in dams or neonates therefore represents a promising strategy to enhance cold tolerance and improve survival during early life. From a fundamental perspective, the rapid postnatal whitening and functional attenuation of BAT in ruminants contrasts sharply with patterns observed in rodents and humans, positioning ruminants as a valuable model for studying adipose tissue plasticity and the developmental programming of energy metabolism (21, 22). Accordingly, this review systematically summarizes recent advances in BAT distribution, function, and regulatory mechanisms in ruminants, highlighting both nutritional modulation and emerging insights into newborn BAT, to provide a conceptual framework for future research and practical applications. However, it should be noted that studies on BAT in small ruminants (e.g., sheep and goats) remain relatively limited compared with cattle, and much of the current understanding is derived from neonatal models.

## 2 Research status of BAT in ruminants

### 2.1 Physiological functions of BAT

BAT–mediated NST represents the most classical and fundamental physiological function of BAT. This process is executed primarily by *UCP1*, which is located in the inner mitochondrial membrane. Under normal conditions, the proton gradient generated by the mitochondrial respiratory chain is coupled to ATP synthesis through oxidative phosphorylation. In contrast, *UCP1* functions as a proton channel that uncouples this process, dissipating the proton motive force directly as heat rather than producing ATP (23). The activation of BAT thermogenesis is initiated by cold exposure. When animals are exposed to low ambient temperatures, thermal signals are perceived by the central nervous system, leading to activation of the sympathetic nervous system (SNS). Norepinephrine (NE) released from sympathetic nerve terminals binds to β3-adrenergic receptors (β3-AR) on the surface of brown adipocytes. Subsequent receptor activation stimulates the G protein–adenylate cyclase signaling cascade, resulting in elevated intracellular cyclic adenosine monophosphate (cAMP) levels and activation of protein kinase A (PKA) (5, 24). Activated PKA triggers two major downstream events. First, PKA phosphorylates and activates hormone-sensitive lipase (HSL), promoting the hydrolysis of intracellular triglycerides (TG) and the release of free fatty acids (FFAs) (25). These FFAs serve a dual role: they act as substrates for mitochondrial β-oxidation to fuel heat production, and they directly activate *UCP1* as allosteric ligands, thereby enhancing proton conductance (5, 26). Second, PKA phosphorylates transcription factors such as cAMP response element-binding protein (CREB), leading to upregulation of *UCP1* and other thermogenesis-related genes, including *PGC-1*α and *Dio2*, thus reinforcing thermogenic capacity at the transcriptional level. This rapid and efficient response is critical for neonatal ruminants to adapt to postnatal cold stress. Beyond thermogenesis, BAT also contributes to systemic metabolic regulation by modulating lipid and glucose metabolism, thereby exerting broader effects on whole-body energy homeostasis and health (26). Collectively, these findings indicate that BAT functions not only as a thermogenic organ but also as a multifunctional metabolic hub in ruminants, warranting further investigation.

Notably, studies on ruminant species adapted to extreme cold environments provide unique insights into the evolutionary significance and regulatory mechanisms of BAT. Musk ox (*Ovibos moschatus*) and reindeer (*Rangifer tarandus*) are the only ruminant species naturally adapted to Arctic conditions. Comparative genomic analyses have revealed that genes undergoing rapid evolution or positive selection in these species are significantly enriched in pathways related to BAT thermogenesis and circadian rhythm regulation (27). For example, a convergent amino acid substitution has been identified in the angiogenesis-related gene HIF2A, which weakens its interaction with prolyl hydroxylase domain protein 2 (PHD2). This alteration may enhance HIF2A stability, thereby promoting vascular endothelial growth factor (VEGF) expression and optimizing blood supply and thermogenic efficiency in BAT (24). In addition, genes involved in circadian rhythm pathways also exhibit signatures of selection, potentially facilitating adaptation to the extreme photoperiods of Arctic environments. These findings highlight the central role of BAT in ruminant cold adaptation and suggest a potential link between thermogenesis and biological clock regulation.

In ruminants, BAT-mediated metabolic regulation may also influence energy utilization efficiency and nutrient partitioning. Active BAT consumes substantial amounts of energy (13), which may negatively affect growth performance and feed efficiency. However, moderate activation of BAT may confer long-term benefits by improving metabolic health. For instance, supplementation of carnitine during late gestation in ewes has been shown to enhance the thermogenic capacity of BAT in neonatal lambs, thereby improving body temperature maintenance and survival under cold conditions (28). Mechanistically, carnitine appears to promote triglyceride and glycogen deposition in brown adipocytes via activation of AMP-activated protein kinase α (AMPKα), thus supporting sustained thermogenesis. These findings provide both theoretical and practical support for nutritional strategies aimed at improving neonatal cold tolerance through BAT regulation.

#### 2.1.1 Distribution and developmental characteristics of BAT

**Figure 1:**
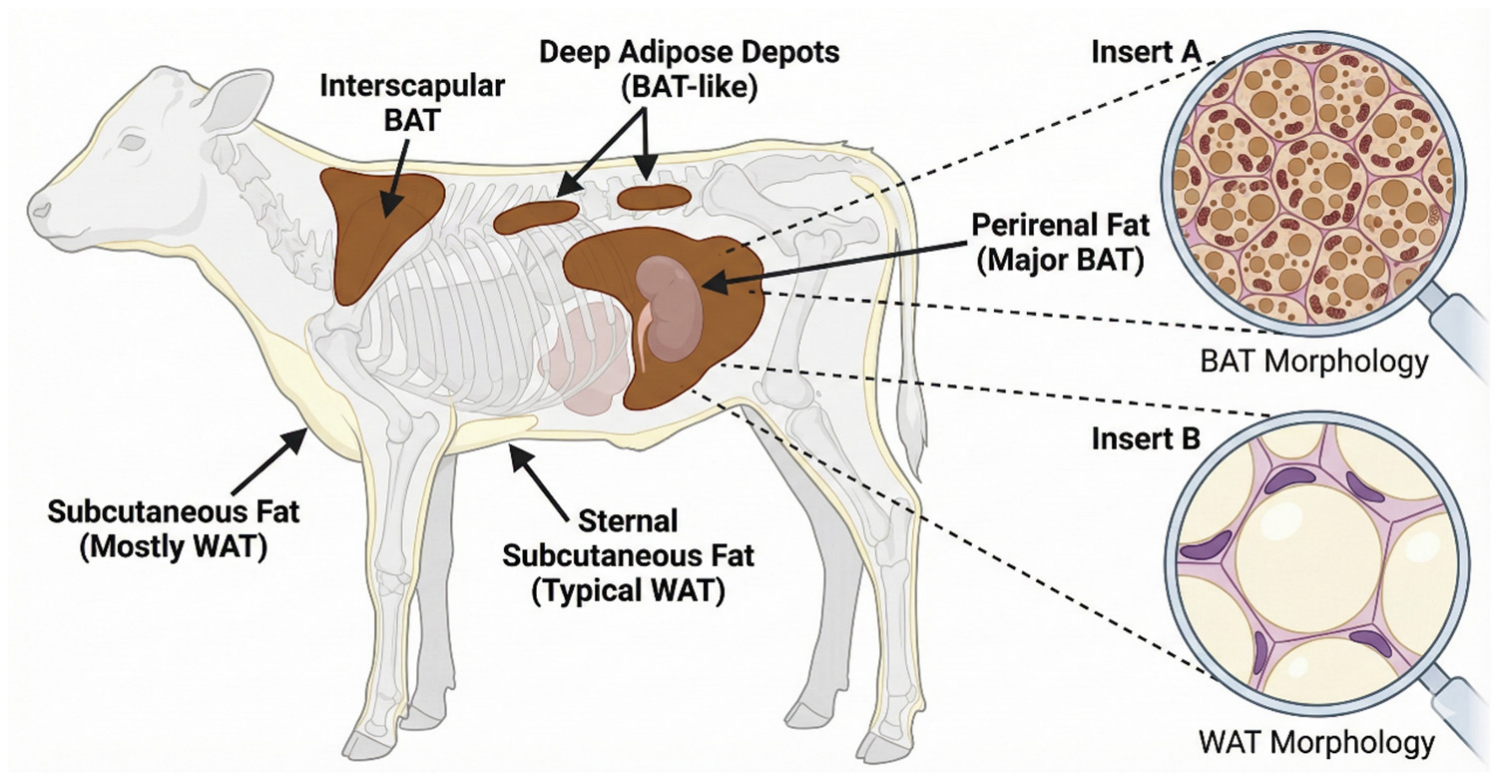
Adipose tissue distribution and morphology in neonatal dairy calves.

In neonatal ruminants, such as dairy cows, BAT is primarily distributed in anatomically strategic regions (Figure 1), including the interscapular and perirenal depots, which are enriched with abundant mitochondria and multilocular lipid droplets to support efficient thermogenesis (6). Similar distribution patterns have also been reported in other ruminants, such as lambs, where BAT is mainly located in the perirenal and prescapular regions (14). The morphology and developmental status of adipose tissue vary across anatomical locations. In neonatal calves, perirenal BAT is particularly abundant and exhibits classic brown adipocyte characteristics, such as multilocular lipid droplets and dense mitochondrial content, consistent with its prominent NST function (6). A similarly prominent perirenal BAT depot has also been reported in neonatal lambs, where it represents a major site of thermogenesis after birth (14). Previous studies have demonstrated a strong negative correlation between age and BAT abundance, with age being the most influential factor determining BAT content (29). In neonatal ruminants, particularly in calves, the majority of adipose depots display cellular features characteristic of BAT, whereas subcutaneous adipose tissue tends to exhibit a white adipose phenotype (30). Specifically, sternal subcutaneous fat in neonatal calves resembles white adipose tissue, whereas caudal (tailhead) subcutaneous fat in both Angus and Brahman calves contains a substantial proportion of brown-like adipocytes. These observations suggest that bovine subcutaneous adipose tissue initially develops as BAT and progressively undergoes postnatal whitening after birth (13). Similar developmental transitions have also been reported in other ruminants. In lambs and goat kids, perirenal BAT undergoes rapid postnatal whitening, gradually acquiring white adipose characteristics (31, 32).

In large ruminants such as cattle and sheep, BAT depots are rapidly recruited after birth, supporting early postnatal growth and energy balance (33). This distinct distribution pattern underscores the functional importance of BAT during the neonatal period and provides a foundation for understanding its subsequent developmental remodeling. Although it has long been assumed that BAT thermogenic activity declines or disappears with advancing age due to postnatal whitening, recent evidence challenges this traditional view. Under prolonged cold exposure or specific nutritional interventions, thermogenically competent beige adipocytes can emerge within white adipose tissue, a phenomenon known as adipose tissue browning (8, 16, 17), highlighting the underestimated plasticity of ruminant adipose tissue. Supporting this notion, increased expression of genes associated with beige adipocyte formation and thermogenesis has been observed in subcutaneous adipose tissue of 34-month-old Mongolian cattle (34). Although direct evidence in sheep or goats is limited, similar browning mechanisms may occur in other ruminants in response to cold exposure and metabolic demand.

### 2.2 Regulatory strategies of BAT in ruminants

**Figure 2:**
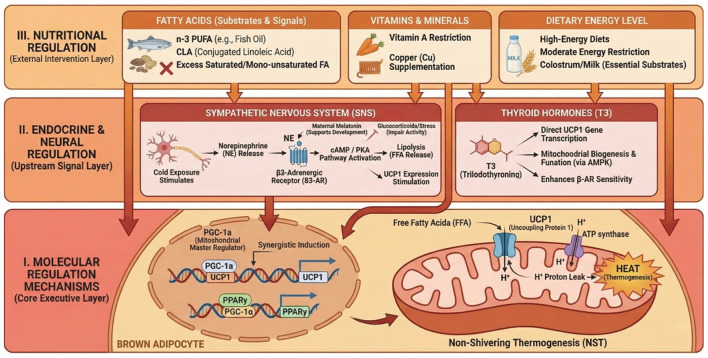
Multilevel regulatory mechanisms of brown adipose tissue thermogenesis in ruminants.

BAT thermogenesis in ruminants is governed by coordinated molecular, endocrine–neural, and nutritional regulatory strategies, which collectively determine the efficiency of non-shivering thermogenesis (Figure 2).

#### 2.2.1 Molecular regulatory mechanisms

BAT thermogenesis is mediated by multiple mechanisms, among which *UCP1*, PGC-1α, and *PPAR*γ are key regulators. *UCP1* is localized in the mitochondrial inner membrane of BAT and is activated by free fatty acids. Upon activation, *UCP1* facilitates proton transport across the inner membrane into the mitochondrial matrix, thereby uncoupling oxidative phosphorylation and dissipating energy as heat (5). This process is essential not only for maintaining body temperature but also for regulating whole-body energy homeostasis and preventing metabolic disorders. The expression of *UCP1* is tightly regulated by multiple stimuli. Cold exposure or β3-adrenergic stimulation induces browning of WAT, thereby promoting *UCP1* expression in beige adipocytes (35–37). In ruminants, *UCP1* expression exhibits pronounced spatiotemporal specificity, emerging during late fetal development, peaking at birth, and rapidly declining under thermoneutral conditions (21, 38). In addition to transcriptional regulation, adequate fatty acid availability is a prerequisite for *UCP1* activation. Specific fatty acids, particularly n-3 PUFA and CLA, have been shown to enhance BAT thermogenic capacity by modulating *UCP1* expression and activity (39). Collectively, *UCP1* serves as both a functional hallmark of BAT and a critical target for investigating energy metabolism.

*PGC-1*α and *PPAR*γ are central transcriptional regulators governing brown adipocyte differentiation, functional maintenance, and thermogenic gene expression. As a master regulator of mitochondrial biogenesis and oxidative metabolism, *PGC-1*α cooperates with *PPAR*γ to induce UCP1 and other thermogenesis-related genes. Moreover, PGC-1α promotes oxidative phosphorylation and fatty acid oxidation, thereby amplifying BAT thermogenic capacity. *PPAR*γ, a key driver of adipogenesis, independently regulates a suite of lipid metabolism genes and specifically facilitates UCP1 induction in BAT (40). Notably, an iBAT-specific enhancer (*Ppar*α*-En4*) has been identified and shown to be activated by cold exposure (33). In addition to PGC-1α and *PPAR*γ, other transcription factors—including *BMP4, BMP7, C/EBP*α, and *PRDM16*—have been reported to regulate brown adipocyte differentiation and thermogenic programming in ruminants (41). The coordinated actions of these factors provide a mechanistic basis for understanding BAT development and function in ruminants.

#### 2.2.2 Endocrine and neural regulation

##### 2.2.2.1 Regulation by thyroid hormones

Thyroid hormones, particularly T3, are pivotal regulators of basal metabolic rate and adaptive thermogenesis, exerting profound effects on BAT development, maintenance, and *UCP1* expression. The primary mechanism involves direct transcriptional regulation through binding of thyroid hormone receptors to response elements in the *UCP1* promoter, thereby enhancing *UCP1* mRNA transcription (42). Beyond direct transcriptional control, thyroid hormones activate the AMPK signaling pathway, promoting mitochondrial biogenesis and improving mitochondrial function (40). Concurrently, thyroid hormones upregulate enzymes involved in fatty acid β-oxidation, ensuring an adequate supply of substrates for *UCP1*-mediated proton leak and heat production (23). Through coordinated regulation of mitochondrial capacity and substrate availability, thyroid hormones enhance BAT thermogenic efficiency at both energy-producing and energy-dissipating levels. Thyroid hormone signaling also interacts extensively with the sympathetic nervous system. T3 enhances β-adrenergic sensitivity by upregulating β1-adrenergic receptors and modulating G-protein signaling components (42). In addition, cross-talk between thyroid hormone receptors, PPAR signaling, and circadian clock genes such as *Per2* suggests an integrated regulatory node linking circadian rhythm, metabolism, and thermogenesis (43).

Overall, thyroid hormones regulate BAT thermogenesis through multilayered mechanisms involving direct gene activation, mitochondrial remodeling, substrate metabolism, and neuroendocrine interactions, highlighting their potential as nutritional or therapeutic targets.

##### 2.2.2.2 Regulation by the sympathetic nervous system

Sympathetic activation induces BAT thermogenesis primarily via NE release, which activates β-adrenergic receptors and downstream cAMP/PKA signaling, thereby stimulating *UCP1* expression and lipolysis (5). Under cold exposure, this pathway initiates mitochondrial uncoupling and activates NST in BAT (23). β-adrenergic signaling also promotes WAT browning through mTORC1 activation, which is required for β-adrenergic-dependent *UCP1* induction and beige/brown adipocyte expansion (44). Central regulation further modulates this process, as stimulation or lesion of the VMH respectively enhances or suppresses BAT thermogenesis (45). Thus, sympathetic regulation represents a rapid and dominant mechanism controlling BAT activity.

The sympathetic–adrenergic system interacts with multiple hormonal and physiological axes. Thyroid hormones potentiate β-adrenergic signaling, whereas chronic stress or elevated glucocorticoids may impair BAT activity by inducing insulin resistance. Circadian regulation of sympathetic output confers diurnal variation in BAT responsiveness to cold stimuli (43). During the perinatal period, maternal melatonin is essential for normal BAT development, as melatonin deprivation reduces BAT mass, abolishes NE-induced thermogenesis, and disrupts expression of thermogenic and adipogenic genes such as *UCP1* and *PPAR*γ (43).

In summary, sympathetic activation via NE release and cAMP/PKA signaling constitutes the core mechanism initiating BAT thermogenesis, while extensive cross-talk with endocrine and circadian systems ensures dynamic regulation of energy balance and thermal homeostasis.

#### 2.2.3 Nutritional regulation

##### 2.2.3.1 Fatty acids

FA serve not only as essential substrates for BAT thermogenesis but also as signaling molecules regulating BAT development and function. PUFA, particularly n-3 PUFA, exert pronounced effects on BAT activity. Dietary supplementation with n-3 PUFA in ruminants has been shown to modulate lipid metabolism and influence BAT development and thermogenic capacity (46). In contrast, excessive supplementation with saturated fatty acids or MUFA during late gestation reduces thermogenic capacity in neonatal lambs, whereas balanced n-6 and n-3 PUFA intake supports BAT function (47). Conjugated linoleic acid (CLA) is a naturally occurring fatty acid found in ruminant products, which can regulate brown adipose tissue (BAT) activity by activating PPARγ and promoting UCP1 expression (48). Nutritional strategies aimed at enhancing CLA bioavailability may improve BAT thermogenic function. Studies have shown that CLA upregulates PPARγ expression and promotes lipid droplet formation in bovine intramuscular preadipocytes, indicating its important role in adipocyte differentiation (49). In mouse models, CLA supplementation has been reported to reduce the mass of both white and brown adipose tissues, accompanied by alterations in the expression of genes and proteins involved in energy metabolism of adipose tissues and skeletal muscle, such as PPAR and UCP2 (50). However, the specific effects of CLA on BAT are influenced by multiple factors, including isomer composition, dosage, species, and physiological status, and thus further studies are warranted.

##### 2.2.3.2 Vitamins and minerals

Vitamins and minerals play indispensable roles in BAT metabolism and functional maintenance, with vitamin A and trace elements being particularly important. Dietary vitamin A restriction has been shown to upregulate BAT- and WAT-related gene expression in cattle adipose tissue, potentially facilitating WAT browning (15, 51). Maternal copper supplementation during gestation increases *UCP1* expression and transiently enhances BAT thermogenesis in neonatal lambs (52). Conversely, inadequate maternal copper intake reduces NE turnover, plasma T3 levels, and cold tolerance in offspring, even in the absence of clinical deficiency (53). These findings underscore the importance of micronutrient adequacy for neuroendocrine regulation of BAT function.

##### 2.2.3.3 Dietary energy level

In ruminants, both maternal nutrition during gestation and postnatal dietary energy intake critically influence the development, maturation, and thermogenic function of BAT. Maternal nutrient restriction or overnutrition during late gestation can impair fetal BAT development by downregulating key transcriptional regulators and thermogenic genes, such as UCP1, PPARγ, C/EBPα, BMP4, BMP7, and PGC-1α, with depot-specific sensitivity observed in perirenal vs. pericardial adipose tissue (54–56). Postnatal energy supply further modulates BAT activity: in neonates and suckling ruminants, energy-dense colostrum and milk provide essential substrates, including fatty acids and glucose, necessary for sustaining thermogenesis (6, 57). At the same time, dietary energy levels can directly regulate BAT metabolic capacity—high-energy diets enhance mitochondrial biogenesis and oxidative function via AMPK activation, whereas chronic overnutrition may suppress BAT activity through excessive fat accumulation, and moderate energy restriction can stimulate lipolysis and fatty acid oxidation, while severe restriction compromises growth and health (5, 58, 59). Collectively, these findings highlight the importance of both prenatal and postnatal nutritional environments in shaping BAT developmental trajectory, thermogenic capacity, and early survival, underscoring the need for carefully balanced energy provision to optimize BAT functionality in ruminants.

## 3 Applications and future research directions

Neonatal hypothermia is a major cause of mortality in neonatal ruminants, particularly in calves and lambs born during winter or early spring (6, 60). In cold regions of northern China and other seasonal calving or lambing systems, nutritional strategies aimed at optimizing BAT function in neonates have direct practical relevance. For instance, maternal supplementation with PUFA-rich diets may enhance offspring BAT thermogenic capacity and cold tolerance by increasing sympathetic activity, elevating circulating T_3_ levels, or suppressing apoptosis in BAT (53). In addition, the development of reliable biomarkers for rapid assessment of BAT reserves or activity in neonates would facilitate early identification of vulnerable animals, enabling targeted management interventions and reducing mortality risk (61).

Beyond cold adaptation, maintaining or reactivating BAT activity may confer long-term metabolic benefits in ruminants. Given its high energy-dissipating capacity, BAT can improve energy balance by increasing resting energy expenditure and limiting excessive lipid deposition. This property suggests potential applications in improving carcass quality in meat-producing animals and in mitigating periparturient metabolic disorders in dairy ruminants, such as ketosis and hepatic steatosis (62). Achieving these outcomes will require the development of strategies to induce white adipose tissue (WAT) browning or to reactivate residual BAT during later growth stages or adulthood.

Future research should leverage multi-omics approaches, including transcriptomics, proteomics, metabolomics, and lipidomics, to elucidate the molecular networks through which nutritional interventions regulate BAT development and function. For example, RNA-seq analyses have shown that cold exposure markedly upregulates thermogenesis-related genes such as *UCP1, PGC-1*α, and *ND1* in goat BAT (63). Lipidomic studies further reveal that short-term cold exposure induces substantial remodeling of triglycerides, glycerophospholipids, and sphingolipids in lamb BAT and plasma (61). Moreover, transcriptomic profiling under specific nutritional interventions, such as n-3 PUFA supplementation, can help identify key regulatory pathways and molecular targets. Integration of multi-omics datasets will enable a more comprehensive understanding of the regulatory cascade linking nutritional signals to BAT thermogenic phenotypes, thereby facilitating the development of targeted intervention strategies. Despite these advances, current multi-omics studies in ruminant BAT remain limited and species-specific, highlighting the need for more integrative and comparative approaches.

## 4 Conclusion

BAT plays a crucial role in energy metabolism and thermoregulation in ruminants, especially during the neonatal period. As the primary organ responsible for NST, it is vital for cold defense and early survival. Beyond this immediate function, its high metabolic activity suggests a broader influence on whole- body energy allocation and metabolic health across the lifespan. Nutritional regulation represents a non-invasive and highly feasible intervention strategy for modulating BAT development, activity, and function by altering fatty acid composition, key nutrient supply, and overall energy intake. At present, research on nutritional regulation of BAT in ruminants remains in its infancy, with many conclusions extrapolated from rodent models. Direct validation and mechanistic investigation in ruminant species are therefore urgently needed.

Future studies should integrate multi-omics approaches with production-oriented nutritional experiments to elucidate ruminant-specific molecular mechanisms underlying nutrition, BAT interactions, while simultaneously evaluating their impacts on production performance, health outcomes, and economic efficiency under practical farming conditions. Such efforts will facilitate the translation of fundamental BAT biology into innovative nutritional strategies, thereby providing new scientific support for the sustainable development of ruminant production systems.
